# What leads to medication errors in polish hospitals from the perspectives of nurses? a multicenter cross-sectional survey

**DOI:** 10.3389/fphar.2026.1824478

**Published:** 2026-06-05

**Authors:** Katarzyna Kwiecień-Jaguś, Monika Kopeć, Anna Małecka-Dubiela, Beata Guzak

**Affiliations:** 1 Department of Anesthesiology Nursing and Intensive Care, Faculty of Health Sciences, Medical University of Gdansk, Gdańsk, Poland; 2 Department of Human Nutrition, University Warmia and Mazury, Olsztyn, Poland; 3 Department of Internal and Pediatric Nursing, Medical University of Gdańsk, Gdańsk, Poland; 4 Department of Nursing and Other Proffesions, School of Public Health, Center of Postgraduate Medical Education, Warsaw, Poland

**Keywords:** hospital units, intensive care unit, internal medicine, medical errors, nurse specialists, nursing research, organisation and administration, patient safety

## Abstract

**Background:**

Patient safety in pharmacotherapy is a critical element of proper medical care, and when properly implemented, it delivers real therapeutic benefits. The project aimed to analyse the causes of medication administration errors in hospitals with varying referral levels. The study was part of a larger research project.

**Methods:**

From the 585 hospitals in Poland, 50 were randomly selected. The 488 nurses agreed to participate in the study. Four hundred sixty-eight completed questionnaires were finally included in the statistical analysis. Research data collection began in 2023, and the entire process took a year. A descriptive analysis for socio-demographic data was used. Factor analysis and varimax rotation were used to transform large amounts of raw, complex data into a clear, interpretable structural model.

**Results:**

The Polish version of the Medication Administration Error scale was validated and rechecked with the alpha–Cronbach index. A part A of the questionnaire, which consists 29 statements about the reason of Medication Administration Errors, achieved 0.93 – with is considered as a very good. The main results showed that the most common reasons for medication error in the administration process in intensive and internal units are: appearance of drug, drug similarity, and illegible orders. On an equal footing in terms of the number of points obtained (AVG > 4), other identified factors are: Pharmacists are not available 24 h a day” (item 12) and “Brand-name drugs are replaced by other generics (item 13). A huge problem is the lack of training on new drugs used in the hospital, and the frequent use of abbreviations. The second, no less important issue, especially in the more conservative units, is interruption in the preparation process and staff shortages.

**Conclusion:**

Most of the factors that lead to MAE, as identified by nursing personnel in internal and intensive care units, can be modified through management processes using prepared tools, scientific recommendations, and technologies. Some areas, such as the shortage of medical personnel in conservative general wards, require further work and up-to-date legislation.

## 1 Introduction

Patient safety in pharmacotherapy is vital for effective healthcare, maximising treatment benefits while reducing adverse drug reactions. Proper drug management includes prescribing, dosing, preparation, and administration by the correct route. Errors can occur at any stage, from prescribing to administration. The final stage—giving the drug to the patient—is especially critical and directly affects patient health. Nurses are responsible for preparing and administering medicines, often while managing other demanding patient care tasks (Bişkin Çetin and Cebeci, 2021).

Statistics show that medication administration errors occur across all medical facilities, including primary care settings, nursing and care facilities, and hospital wards (Elliott et al., 2021). However, some researchers indicate that highly specialised units, such as intensive care or paediatrics, may experience such incidents much more frequently (Juneja and Mishra, 2022). General data on the reporting of medication administration events and errors are quite varied. According to the World Health Organisation (WHO), 4 out of 10 patients experience harm in primary and outpatient medical care settings. Studies confirm that 80% of situations dangerous to the patient can be avoided (WHO). Other analyses conducted in the United States show that medication errors are the third leading cause of death in the American population, and that a significant proportion of patient deaths is not captured by the ICD code but results from human, organisational, and systemic errors (Makary and Daniel, 2016). The current state of knowledge in Poland regarding medication administration errors and medical errors is characterised by a significant information gap. While both Western European countries and the United States have extensive reporting and event analysis systems, the Polish healthcare system relies on highly fragmented data and limited transparency. This may be attributed to a shortage of appropriate monitoring tools, as well as general regulations that, under the current legal framework, grant healthcare facilities the freedom to choose their own monitoring systems for such incidents.

The first pilot study about medication errors among Polish nurses was published in 2022 (Kwiecień-Jaguś et al., 2022). The research question concerned the analysis of the causes of errors or adverse events in medication administration. The study design, including the survey questionnaire, was based on the research conducted by Sanghera (Sanghera et al., 2007). These very preliminary analyses showed that medical personnel identified the following as the primary factors leading to errors in the medication administration process: inadequate staffing levels during shifts, significant overload and stress, and the similarity of medication labels (look-alike drug labelling). Most respondents declared that when such incidents occur, the majority of situations are reported to a supervisor or the attending physician (Kwiecień-Jaguś et al., 2022). Regarding the most common types of errors, the lack of available domestic research necessitates a reliance on international sources. The most frequent errors associated with the medication administration process involve: administering the wrong medication, incorrect dosage, improper preparation, incorrect route of administration, failure to administer the drug or omission of a dose, and incorrect timing of administration (Jessurun et al., 2023; Brabcová et al., 2023).

This research project represents a pioneering attempt at a multifaceted analysis of the root causes of medication administration errors across both internal and highly specialised hospital wards in Poland. By addressing the current data deficit, this study establishes a foundation for evidence-based interventions to strengthen the culture of patient safety. Ultimately, these findings may serve as a critical catalyst for the development of standardised prevention protocols and comprehensive monitoring systems.

The research project being conducted was the first project of that kind on a national scale in Poland. The aim of the study was to examine the reasons for medication administration errors (MAEs) in hospitals across different reference levels and unit types.

## 2 Methods

### 2.1 Study design

A descriptive cross-sectional design was used in that study. The STROBE guideline for cross-sectional studies was used.

### 2.2 Data

The research was conducted from 1 May 2023, to 30 December 2024, following approval from the Bioethics Committee to use the standardised tool, the Medication Administration Error Survey (MAEs) (Wakefield et al., 2005), developed by Prof. B. Wakefield. The researchers obtained the author’s consent to translate the original tool into Polish and to introduce minor changes to address existing cultural and organisational differences. The scale’s reliability was assessed using Cronbach’s alpha and an exploratory factor analysis in a separate pilot study. The results of the pilot studies validating the tool were published in 2025 (Kwiecień-Jaguś et al., 2025). The internal consistency of the Polish tool was within acceptable limits. Given the wide range of data collected from many medical facilities with different organisational structures, the need to narrow the analysis to a key area led the investigators to focus solely on Part A of the survey questionnaire (statements 1–29), which addressed the causes of medication errors on the ward. Cronbach’s alpha for this section was excellent, at 0.93.

The B and C subsections of the questionnaire focus on why medication administration errors are not reported and on the percentage of errors in the units (Wakefield et al., 2005). In sections A and B, respondents marked their responses on a 6-point Likert scale, where 1 = strongly disagree and 6 = completely agree. The subsequent questions in the questionnaire concerned socio-demographic data (Wakefield et al., 2005).

The usefulness of the tool designed by Professor Wakefield was confirmed in many international studies led in the Czech Republic (Brabcová et al., 2023), Turkey (Bişkin Çetin and Cebeci, 2021), Ethiopia (Bifftu et al., 2016), and Jordan (Alzoubi M. M. et al., 2023).

#### 2.2.1 Definition of medication administration error survey

The general definition of MAE, first introduced by Professor B. Wakefield, was translated and included in the Polish version. The definition describes a medication administration error as an error related to the ingestion, injection, or administration of individual therapeutic doses (e.g., incorrect drug, wrong patient, wrong drug, incorrect diluent) (Wakefield et al., 2005).

### 2.3 Participation and research design

A total of 488 nurses employed in Polish hospitals at various referral levels participated in the study. The project encompassed nursing staff from general internal wards as well as those working in the highly specialized field of intensive care. According to data from the Central Register of Nurses and Midwives in Poland, there were 315,670 registered nurses as of January 2023, corresponding to the timing of the study implementation (Chief Chamber of Nurses and). The minimum sample size required to achieve the desired statistical power was calculated using the Sample Size Calculator (Sample Size Calculator). Based on the total number of registered nurses, the optimal sample size was determined to be 384 respondents, providing a 95% confidence level and a 5% margin of error. From a list of 585 hospitals across 16 voivodeships, 50 facilities with different referral levels were randomly selected. Each facility director and Nursing Manager was invited, by official letter, to participate in the research project. 25 facilities refused to participate in the project. Facilities that agreed to conduct the study received the research kit with a return envelope by mail within 2 weeks. The ward nurses of the general and intensive care units, selected by the managers, distributed the questionnaires among their employees. Completing the questionnaire took no more than 10–15 min. Participation of nursing staff from selected departments in the research project was voluntary. All respondents were informed of the analysis’s purpose and their right to withdraw from the study at any time. The project was positively received by those who agreed to participate. Respondents participating in the project, as well as the coordinating nurses and nursing directors, demonstrated a deep understanding of the importance of the issues being addressed, as evidenced by their kindness, reliability, and the few incorrectly completed questionnaires. After collecting the questionnaires at the facility, the package was returned to the principal investigator. The study planned to include 500 respondents. A 97% return rate was achieved. 20 questionnaires from the 488-sample were rejected due to incompleteness.

### 2.4 Hospital characteristics

In Poland, the fundamental hospital security framework for healthcare delivery is organised into a system known as the hospital network. This model was established in 2017 to enhance the organisation of medical services. Hospitals within this network provide patients not only with standard hospital care but also with specialised medical and rehabilitation services tailored to the patient’s health status. Eligibility for inclusion in the hospital network is reviewed and renewed every 4 years. Hospitals are stratified into hierarchical levels, ranging from basic medical procedures to highly complex, specialised treatments. This stratification defines three primary referral tiers: the first includes district hospitals, which deliver essential local healthcare; the second comprises voivodeship (provincial) hospitals capable of providing more advanced medical interventions; and the third, designated as the highest national level, encompasses research institutes and clinical hospitals equipped to manage severe cases and utilise the most advanced medical technologies. The current list of designated hospitals for the period 2023–2027 comprises 585 institutions across 16 voivodeships. Inclusion on this list ensures ongoing funding and operational stability, contingent upon agreements with the National Health Fund (National Health Found). It is important to note that specific operational standards for various hospital units, such as intensive care units (ICUs), are governed by separate legal regulations, which specify, among other things, the minimum nursing staff per patient and detailed unit characteristics. According to the Polish Anaesthesiology and Intensive Care Unit Association, ICUs treat patients with acute conditions involving multi-organ or system failure who require advanced and specialised therapeutic techniques (Polish Society of Anaesthesiology and Intensive Care, 2022; Regulation of the Minister of Health, 2016). Medication use in ICUs is the cornerstone of care. Many of them are classified as High Alert Medications, which require stringent safety protocols (Clerk, 2023). Conversely, internal medicine units are primarily focused on diagnostic processes and the management of patients with stable, chronic conditions. Internal medicine departments, depending on the hospital, may often have a very narrow degree of specialisation, including diabetology, cardiology, nephrology, pulmonology, or general internal medicine (Act on Medical Activity, 2011; Announcement of the Minister of).

Both types of units are present in hospitals from the first to the third referral levels, but of course they differ from each other according to the type of patients, organisation, and size and amount of qualified staff.

### 2.5 Inclusion and exclusion criteria

#### 2.5.1 Inclusion criteria

- Nurses worked in inpatient units (internal and ICU) in selected hospitals with different reference levels in Poland
- Informed consent

#### 2.5.2 Exclusion criteria

- Other medical specialists like doctors, pharmacists, paramedics
- Nursing Managers of the Hospitals

### 2.6 Statistical analysis

A descriptive analysis was initially performed in the statistical studies. Of the 488 questionnaires, 4% were excluded from the entire group due to the missing data. The remaining few missing answers were supplemented with the mode value of the given variable. Then, a factor analysis was performed to identify hidden factors from the data. Due to the extensive nature of the study and the many variables, socio-demographic data, including gender, age, level of education, and hospital referral level, as well as respondents’ answers to questions 1–29 of the survey, were included in the analysis. Two factors were identified using Cattell’s graphical criterion (scree plot). The use of varimax rotation increased the reliability of interpreting results by assigning specific variables to specific axes.

Although socio-demographic factors were significant in the one-way analysis of variance, they did not differentiate the groups of respondents in the factor analysis based on responses to subsequent survey questions (1–29).

### 2.7 Ethical approval

This study was conducted in accordance with the Declaration of Helsinki (WMA Declaration of Helsinki Ethical), as updated and approved by the Ethics Research Committee of the Medical University (NKBBN/185/2023) on 26 April 2023.

## 3 Results

Questionnaires from 468 respondents were included in the statistical analysis.

### 3.1 Sociodemographic sample characteristics

**FIGURE 1:**
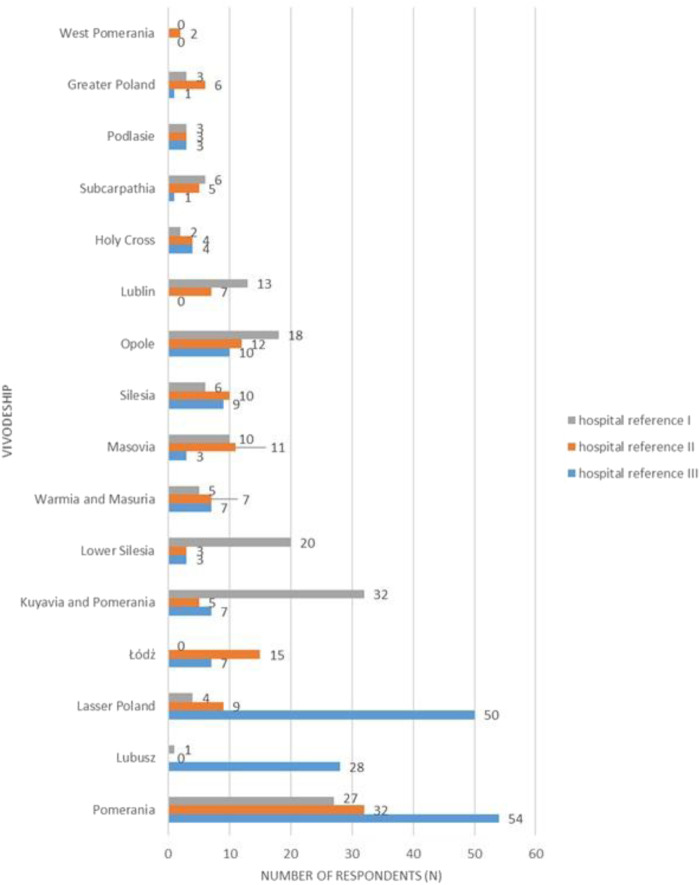
Number of hospitals participating in the project by region of Poland and reference level.

The distribution of hospitals by the level of medical services provided (referral level) was fairly balanced: 150 respondents were from hospitals at the first referral level (32.05%) 131 from the second (27.99%), and 187 (39.96%) from the third. Most respondents worked in hospitals located in Pomerania, Kuyavia and Pomerania, Lesser Poland, and the Opole Region. A detailed analysis of results by referral level and region is presented in Figure 1.

The study group was quite diverse. Among intensive care personnel, the largest group was aged 30–50 years (n = 201, 52%), while in internal wards, this age group accounted for 55% (n = 47) of the respondents. The second-largest group comprised individuals under 30 years old: 27% (n = 105) in intensive care units and 31% (n = 26) in internal wards. The vast majority of respondents in both highly specialised and internal wards were women. Respondents with secondary nursing education still constituted the largest group. A detailed comparison of responses by department is presented in Table 1.

**TABLE 1:** Socio-demographic characteristics of the studied group.

Age	Intensive care N%		Internal unit N%	
<30	105	27%	26	31%
30–50	201	52%	47	55%
>50	77	20%	12	14%
Gender				
Women	356	93%	27	32%
Men	77	20%	8	9%
Hospital references level				
I Reference level	117	31%	33	39%
II references level	104	27%	27	32%
III references level	162	42%	25	29%
Education				
RN	231	60%	60	71%
Bachelor of nursing	99	26%	22	26%
Master of nursing	53	14%	3	4%

N, absolute frequency; % - relative frequency.

**FIGURE 2:**
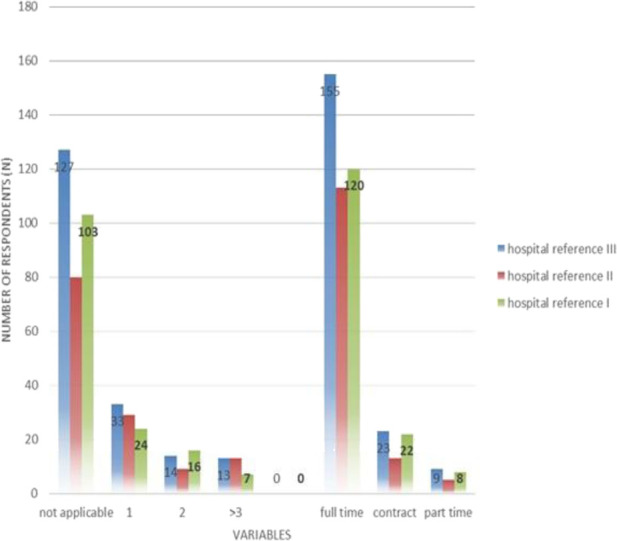
Number of nursing transfers to another unit per year vs. Type of employment.

Analysis of employment status and the number of transfers to different units showed that most employees at referral levels I, II, and III held full-time positions. Fifty-eight respondents worked under contract, and 22 were employed part-time (Figure 2).

A closer look at cross-unit transfers throughout the year revealed that, for the vast majority of cases across all referral levels, such transfers were rare. However, in 13 cases, nurses at the highest referral level were transferred more than three times per year. This issue affected 13 nurses at the second-highest level and 7 at the I level. Detailed summaries of these data are presented in Figure 2.

### 3.2 Reasons for medication administration errors in polish hospitals

A factor analysis was used on the socio-demographic data and a 29-item survey. Based on the scree plot criterion, a two-factor solution was identified, explaining 33.42% of the variance. The application of a Varimax rotation facilitated a more robust interpretation by clarifying the distribution of variable loadings across the identified axes.

Medication administration errors in Polish hospitals are mainly linked to two factors identified by factor analysis: drug - related issues and hospital pharmacy operations. Together, these factors account for 39.5% of the total variance in responses from nursing staff surveyed about 29 possible causes (Likert scale 1–6). The most frequently cited causes were similar drug packaging (items 3 and 4), illegible orders (item 5), limited pharmacist availability (item 12), substitution with generics (item 13), and lack of training on new drugs (item 16). Additional significant issues included staff shortages (item 23), time constraints (item 24), and use of abbreviations (item 7) (Table 2).

**TABLE 2:** Reasons for MAEs–comparison of mean values between intensive care units and general.

The reasons why medication administration error happened in the medical facilities - part A	Intensive care nurses				Internal nurses			
	Mo	Me	AVG	S	Mo	Me	AVG	S
1. The names of many medications are similar	5	4	3.93	1.39	5	4	3.87	1.40
2. Different medications look alike	6	6	4.83	1.47	6	5	4.58	1.48
3. The packaging of many medications is similar	6	6	4.96	1.39	6	6	4.79	1.50
4. Physicians’ medication orders are not legible	6	4	4.07	1.55	6	5	4.32	1.62
5. Physicians’ medication orders are not clear	2	4	3.67	1.38	5	4	3.95	1.44
6. Physicians change orders frequently	4	4	3.80	1.37	4	4	4.08	1.43
7. Abbreviations are used instead of writing the orders out completely	2	3	3.45	1.42	6	4	4.02	1.59
8. Verbal orders are used instead of written orders	2	4	3.57	1.37	2	4	3.65	1.48
9. Pharmacy delivers incorrect doses to this unit	2	2	2.53	1.12	2	2	2.74	1.27
10. Pharmacy does not prepare the med correctly	2	2	2.31	0.93	2	2	2.61	1.30
11. Pharmacy does not label the med correctly	2	2	2.40	1.04	2	2	2.52	1.23
12. Pharmacists are not available 24 h a day	6	4	4.08	1.74	2	3	3.73	1.69
13. Frequent substitution of drugs (i.e., cheaper generic for brand names)	6	4	4.20	1.50	6	4	4.07	1.64
14. Poor communication between nurses and physicians	2	4	3.58	1.35	2	4	3.62	1.43
15. Many patients are on the same or similar medications	4	4	4.21	1.39	4	4	4.02	1.38
16. Unit staff do not receive enough inservices on new medications	6	4	4.18	1.51	6	4	4.18	1.66
17. On this unit, there is no easy way to look up information on medications	2	4	3.61	1.52	4	4	3.71	1.49
18. Nurses on this unit have limited knowledge about medications	2	3	3.17	1.34	2	3	3.41	1.37
19. Nurses get pulled between teams and from other units	2	2	3.17	1.44	2	4	3.71	1.60
20. When scheduled medications are delayed, nurses do not communicate the time when the next dose is due	2	3	3.28	1.42	2	4	3.74	1.57
21. Nurses on this unit do not adhere to the approved medication administration procedure	2	2	2.85	1.31	2	3	3.02	1.41
22. Nurses are interrupted while administering medications to perform other duties	2	4	3.96	1.50	6	5	4.34	1.69
23. Unit staffing levels are inadequate	2	4	3.92	1.58	6	5	4.44	1.64
24. All medications for one team of patients cannot be passed within an accepted time frame	2	4	3.89	1.52	6	4	4.18	1.54
25. Medication orders are not transcribed to the electronic/paper order system correctly	2	3	3.39	1.40	4	4	3.98	1.47
26. Errors are made in the electronic/paper order system	2	3	3.34	1.35	4	4	3.84	1.45
27. Equipment malfunctions or is not set correctly (e.g., IV pump)	2	3	3.14	1.37	2	3	3.32	1.39
28. Nurse is unaware of a known allergy	2	2	2.92	1.28	2	3	3.20	1.37
29. Patients are off the ward for other care	2	2	2.88	1.23	2	3	3.13	1.26

Mo, mode; Me, Median; AVG, average; s, standard deviation.

**FIGURE 3:**
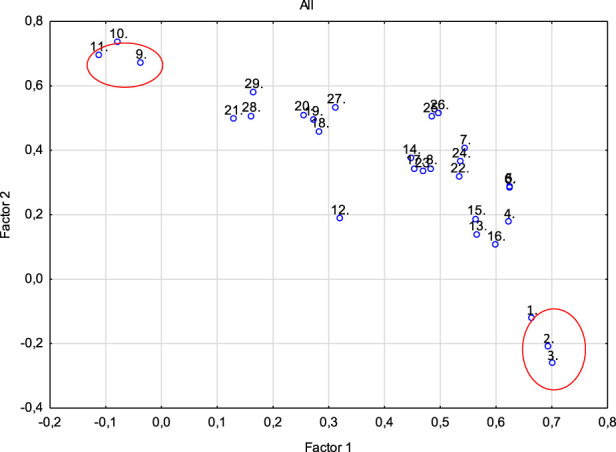
Reasons for medication administration errors - factor analysis.

Unit differences were observed: in general, units reported more pronounced problems, such as staff shortages, inability to transfer all drugs for a patient group on time, and frequent use of abbreviations. For ICU nurses, pharmacy-related issues (Factor 2) were most significant. Among other nurses, both pharmacy-related (Q9–Q11, loadings 0.82–0.85) and drug packaging issues (S1–S3, loadings 0.72–0.76) were highlighted, with support from statement 22 (0.72). Factor 2 remained robust after adjusting for demographics, while Factor 1 (drug-related issues) decreased in significance (0.68). No significant correlation was found between error causes and demographic or organisational factors. Detailed statistics are presented in Tables 2, 3 and Figure 3 and Supplementary Table 1.

**TABLE 3:** Factor analysis of elements having impact on medication errors.

Statements	All		Intensive care nurses		Internal nurses	
	Factor 1	Factor 2	Factor 1	Factor 2	Factor 1	Factor 2
1. The names of many medications are similar	0,664,566	−0,119,730	0,624,936	−0,184,791	0,712,865	0,005,997
2. Different medications look alike	0,694,407	−0,208,032	0,657,839	−0,291,248	0,715,641	0,002,621
3. The packaging of many medications is similar	0,701,648	−0,260,607	0,651,503	−0,335,080	0,750,676	−0,143,455
4. Physicians’ medication orders are not legible	0,624,089	0,179,410	0,637,780	0,111,085	0,585,117	0,282,555
5. Physicians’ medication orders are not clear	0,626,030	0,285,640	0,644,514	0,211,001	0,608,783	0,401,137
6. Physicians change orders frequently	0,624,860	0,283,214	0,637,420	0,227,975	0,664,152	0,284,301
7. Abbreviations are used instead of writing the orders out completely	0,545,182	0,404,612	0,559,500	0,349,134	0,625,461	0,379,373
8. Verbal orders are used instead of written orders	0,484,533	0,343,072	0,510,613	0,290,789	0,458,239	0,457,089
9. Pharmacy delivers incorrect doses to this unit	−0,036,448	0,670,433	−0,027,909	0,647,494	0,066,709	0,826,396
10. Pharmacy does not prepare the med correctly	−0,077,277	0,734,375	−0,073,864	0,711,495	0,044,897	0,854,448
11. Pharmacy does not label the med correctly	−0,112,445	0,693,833	−0,101,759	0,683,230	−0,007,623	0,830,573
12. Pharmacists are not available 24 h a day	0,319,917	0,187,082	0,351,930	0,150,281	0,206,525	0,440,571
13. Frequent substitution of drugs (i.e., cheaper generic for brand names)	0,567,136	0,137,491	0,584,209	0,107,520	0,478,785	0,249,344
14. Poor communication between nurses and physicians	0,448,340	0,374,948	0,449,442	0,355,937	0,598,267	0,266,848
15. Many patients are on the same or similar medications	0,564,580	0,186,059	0,564,527	0,152,075	0,617,082	0,239,291
16. Unit staff do not receive enough inservices on new medications	0,600,312	0,105,581	0,599,389	0,070,396	0,624,685	0,080,819
17. On this unit, there is no easy way to look up information on medications	0,454,527	0,342,249	0,469,493	0,322,970	0,582,730	0,201,529
18. Nurses on this unit have limited knowledge about medications	0,284,156	0,457,113	0,299,807	0,474,257	0,481,798	0,129,833
19. Nurses get pulled between teams and from other units	0,272,940	0,494,017	0,238,833	0,454,065	0,588,246	0,376,783
20. When scheduled medications are delayed, nurses do not communicate the time when the next dose is due	0,255,267	0,509,333	0,231,701	0,501,893	0,565,671	0,275,072
21. Nurses on this unit do not adhere to the approved medication administration procedure	0,129,385	0,496,577	0,126,643	0,485,456	0,330,526	0,395,450
22. Nurses are interrupted while administering medications to perform other duties	0,535,163	0,317,054	0,512,898	0,258,941	0,724,705	0,257,779
23. Unit staffing levels are inadequate	0,470,904	0,333,270	0,483,163	0,249,988	0,578,077	0,348,602
24. All medications for one team of patients cannot be passed within an accepted time frame	0,537,767	0,363,472	0,524,670	0,303,486	0,699,816	0,369,124
25. Medication orders are not transcribed to the electronic/paper order system correctly	0,485,948	0,503,937	0,487,160	0,445,083	0,637,565	0,525,525
26. Errors are made in the electronic/paper order system	0,498,444	0,513,742	0,511,405	0,455,425	0,605,398	0,543,231
27. Equipment malfunctions or is not set correctly (e.g., IV pump)	0,313,543	0,531,901	0,311,270	0,512,432	0,497,991	0,477,619
28. Nurse is unaware of a known allergy	0,160,884	0,506,027	0,159,143	0,497,294	0,417,059	0,295,178
29. Patients are off the ward for other care	0,165,702	0,579,206	0,133,381	0,560,726	0,472,002	0,514,813

## 4 Discussion

The study presents an analysis of the causes of errors in the drug administration process among nursing staff in hospitals at different referral levels in Poland, accounting for ward type. Analysing the collected data shows that the similarity of drug packaging and the illegibility of doctors’ orders are among the most frequent causes of medication administration errors identified by medical personnel.

The analysed results are consistent with those of other scientists (Brabcová et al., 2023; Bifftu et al., 2016; Alzoubi et al., 2023). Look-alike medication and sound-alike medication (Look Alike and Sound Alike - LASA) can lead to overdosing, underdosing or inappropriate dosing of medicines (Bryan et al., 2021). Given the large number of drugs on the market, The Joint Commission recommends that every hospital prepare its own list of LASA drugs for its healthcare service site. This would be a great facilitation for employees and people involved in the drug administration process (The Joint Commision, 2025). Another solution to avoid mistakes is: “do not disturb zones”, storing LASA drugs separately, using 10 R rule in practice, a barcoding system or Tall Man lettering (Palese et al., 2019; Zheng et al., 2021; Grailey et al., 2023). The latest publications recognise the benefits of incorporating AI into the computerised physician order entry system (Chalasani et al., 2023). In relation to the obtained results of our own research, it is necessary that solutions such as safety zones, proper arrangement of drugs in the unit cabinet, clear labelling of drugs, and development of our own LASA lists are implemented into clinical practice, along with the support of modern technologies that reduce the number of errors.

In Poland, access to such solutions remains a facility-level decision, dependent on finances. The lack of a uniform system means that some medical facilities do not implement effective measures to ensure patient safety during drug administration. Data from the Chief Chamber of Nurses and Midwives leaves no illusions: only 33% of nurses and midwives have access to safe equipment that enables proper preparation and administration of drugs (Chief Chamber of Nurses and Midwives, 2023). Despite increasing expenditure on healthcare (Healthcare spending is rising) and current guidelines (national and foreign), progress in safe drug preparation and administration practices in Poland over the last 30 years has been limited. For this reason, it is impossible to compare the results of this study with those of other studies. Our findings indicate that nursing staff are aware of the consequences of medication administration errors (Sanghera et al., 2007). This knowledge is supported by the findings, which underscore the need for changes in this area and the expectations of this professional group. Access to modern technologies and a proper work culture can significantly reduce the incidence of such incidents. Awareness of the percentage of incidents reported in the central registry rather than in media reports, and analysis of orders using AI, are further milestones in improving the situation. Perhaps a better future lies in European funds for the technological modernisation of Polish healthcare. The assumption is that 4.4 billion PLN will be transferred for the changes of Polish medical facilities, including the implementation of electronic patient documentation in all publicly funded hospitals, improvements in working conditions for medical personnel, and greater use of AI (National Reconstruction Plan).

Our research proved a strong correlation between Medication Administration Errors (MAEs) and limited access to hospital pharmacists. Concerns among nursing staff about the diminishing role of pharmacists highlight the urgent need for reform and improved interdisciplinary collaboration. The obtained results proved that this problem appeals not only in the small hospitals with I reference level but also in big and more specialised university centres. Hospital pharmacists are not just medication suppliers; Polish law requires their active involvement in patient care. As autonomous medical professionals, pharmacists in Poland have broad responsibilities under the Act on the Profession of Pharmacist, including ensuring the safety, quality, and appropriate selection of medicines. Their duties also cover storage, ward distribution, and management of medicinal products and special medical foods. They play a key role in preparing daily doses, infusion fluids, and parenteral nutrition. According to Article 4, point 2 of the Act, hospital pharmacists must collaborate with other clinicians to ensure accurate pharmacotherapy, verify dosages, manage drug substitutions, and identify drug interactions (Przepisy, 2020). Available publications confirm that the constant presence and involvement of a hospital pharmacist in the pharmacotherapy process can significantly reduce the incidence of drug administration errors (Naseralallah et al., 2025). However, further research in this area is necessary.

The second issue raised by nursing staff was the illegibility of medical orders and the excessive frequency of changes in recommendations. This posed a significant challenge during the study implementation and tool validation (Kwiecień-Jaguś et al., 2025). The lack of a unified system across the country translates into numerous difficulties. Research shows that standardising systems, continuously improving the user interface, and enhancing tools for both the ordering process and drug distribution are key to reducing potential problems (Sharma et al., 2024; Alharthi and Alshagrawi, 2024; Washington DC et al., 2005).

Another crucial issue is the significant workload and multitasking required of nursing staff. Our analysis clearly showed that nurses working in general internal wards, particularly, experience higher workloads. This is largely attributable to current staffing standards and years of insufficient implementation of organisational solutions, such as hiring support staff to relieve nurses of administrative tasks. Performing professional duties amid constant interruptions results in decreased concentration and reduced ability to complete tasks, including safe medication preparation (Moriwaki et al., 2025). While many nurses in the study held full-time positions, other acceptable forms of employment included contracts in which the employee commits to a negotiated number of hours with the hospital. The findings also revealed that, although not universal, in some hospital units, nurses are seconded to other departments, which can undermine the development of a robust safety culture. Therefore, further research is warranted to examine the effects of extended working hours on medication administration errors, to assess working memory load during nursing activities, and to determine optimal nurse-to-patient ratios.

A key limitation of the project was the use of a questionnaire, which captures only nurses’ subjective opinions on medication error causes and omits deeper analysis of environmental, personal, and organisational factors. It also excludes perspectives from other medical staff involved in the administration process, like doctors and pharmacists. Another research limitation was the selection of the study group. Where the first part of the selection was conducted randomly, the second steep of the selection used convinience based sampling. Nevertheless, that was the only option to reach so many nursing staff, from different medical facilities and different hospital reference levels.

The strengths of this research include its representative sample and its focus on a critical yet often overlooked topic—patient safety in drug management. This study is unique on the Polish scale for its comprehensive investigation of factors contributing to medication errors, addressing a gap in both clinical practice and the academic literature.

## 5 Conclusion

Efforts to reduce medication administration errors should focus on standardising medication ordering systems and monitoring adverse events. It is essential to expand hospital pharmacists’ roles and increase their accessibility to nursing staff, particularly for dosage counselling, drug-drug interactions, and the use of more affordable alternatives.

Improving patient safety cannot be treated as merely an additional task delegated solely to nurse managers; rather, it requires a systemic approach. Through these interventions, the preparation of medication—an act often perceived as simple—will no longer be viewed by nursing staff as a figurative ‘trap'.

## Data Availability

The raw data supporting the conclusions of this article will be made available by the authors, without undue reservation.
