# Supplementary material for: What leads to medication errors in polish hospitals from the perspectives of nurses? a multicenter cross-sectional survey
Source: Front Pharmacol. 2026 Jun 5;17:1824478. doi: 10.3389/fphar.2026.1824478 (PMC13279710; doi:10.3389/fphar.2026.1824478)
Supplement: Supplementary file 1 [file Table1.docx]

Supplementary Table 1. Factor analysis – reasons for MAE vs socio-demographic data (Suppl. Material).

| Socio-demographic variable vs reasons for MAE | All | | Intensive Care Nurses | | Internal nurses | |
| --- | --- | --- | --- | --- | --- | --- |
|  | Factor 1 | Factor 2 | Factor 1 | Factor 2 | Factor 1 | Factor 2 |
| a.Education | -0,010787 | -0,165124 | -0,047681 | -0,151014 | -0,01049 | -0,129999 |
| b.Hospital reference level | -0,158591 | 0,083534 | -0,143161 | 0,184096 | -0,11970 | -0,037994 |
| c. Sex | 0,115097 | -0,047309 | 0,112658 | -0,074662 | -0,19476 | -0,121627 |
| d.Age | 0,105994 | -0,152804 | 0,077326 | -0,169954 | 0,05974 | -0,185265 |
| 1. The names of many medications are similar. | 0,657851 | -0,125471 | 0,589601 | -0,269862 | 0,68123 | -0,172264 |
| 2. Different medications look alike. | 0,681593 | -0,207916 | 0,602868 | -0,370193 | 0,67875 | -0,178648 |
| 3. The packaging of many medications is similar. | 0,684354 | -0,255089 | 0,588268 | -0,408274 | 0,66699 | -0,316376 |
| 4. Physicians' medication orders are not legible. | 0,630718 | 0,164450 | 0,649995 | 0,010310 | 0,63469 | 0,130168 |
| 5. Physicians' medication orders are not clear. | 0,626440 | 0,279485 | 0,665547 | 0,117883 | 0,68832 | 0,238540 |
| 6. Physicians change orders frequently. | 0,625697 | 0,275072 | 0,659855 | 0,137577 | 0,72358 | 0,050318 |
| 7. Abbreviations are used instead of writing the orders out completely. | 0,553733 | 0,391880 | 0,606995 | 0,254809 | 0,70135 | 0,200402 |
| 8. Verbal orders are used instead of written orders. | 0,490923 | 0,333794 | 0,546716 | 0,213747 | 0,57307 | 0,302057 |
| 9. Pharmacy delivers incorrect doses to this unit | -0,017206 | 0,659467 | 0,074062 | 0,627280 | 0,30723 | 0,743417 |
| 10. . Pharmacy does not prepare the med correctly. | -0,056064 | 0,724666 | 0,038977 | 0,698220 | 0,29516 | 0,775442 |
| 11. Pharmacy does not label the med correctly. | -0,093591 | 0,686209 | 0,007179 | 0,673339 | 0,22314 | 0,815893 |
| 12. Pharmacists are not available 24 hours a day. | 0,350116 | 0,145355 | 0,390022 | 0,054664 | 0,32828 | 0,351126 |
| 13. Frequent substitution of drugs (i.e., cheaper generic for brand names). | 0,574324 | 0,121736 | 0,598327 | 0,011766 | 0,51848 | 0,142573 |
| 14. Poor communication between nurses and physicians. | 0,452679 | 0,368255 | 0,494298 | 0,289552 | 0,65356 | 0,078719 |
| 15. Many patients are on the same or similar medications. | 0,577169 | 0,163050 | 0,589588 | 0,048842 | 0,65549 | 0,067079 |
| 16. Unit staff do not receive enough inservices on new medications. | 0,609918 | 0,085853 | 0,607983 | -0,026571 | 0,62498 | -0,106002 |
| 17. On this unit, there is no easy way to look up information on medications. | 0,469729 | 0,323488 | 0,519591 | 0,235397 | 0,61335 | 0,041572 |
| 18. Nurses on this unit have limited knowledge about medications. | 0,292476 | 0,451655 | 0,364732 | 0,427839 | 0,51052 | -0,046113 |
| 19. Nurses get pulled between teams and from other units. | 0,292367 | 0,476282 | 0,310871 | 0,398997 | 0,67709 | 0,179380 |
| 20. When scheduled medications are delayed, nurses do not communicate the time when the next dose is due. | 0,263207 | 0,506186 | 0,297742 | 0,471853 | 0,63689 | 0,052807 |
| 21. Nurses on this unit do not adhere to the approved medication administration procedure. | 0,136742 | 0,496047 | 0,195649 | 0,463051 | 0,43002 | 0,266964 |
| 22. Nurses are interrupted while administering medications to perform other duties. | 0,527029 | 0,325857 | 0,532566 | 0,208600 | 0,76965 | 0,040815 |
| 23. Unit staffing levels are inadequate. | 0,471600 | 0,333867 | 0,508009 | 0,194329 | 0,66236 | 0,135585 |
| 24. All medications for one team of patients cannot be passed within an accepted time frame. | 0,537505 | 0,360931 | 0,555715 | 0,237926 | 0,77607 | 0,155993 |
| 25. Medication orders are not transcribed to the transcribed to the electronic /paper order system correctly. | 0,488730 | 0,502701 | 0,542145 | 0,378785 | 0,76345 | 0,312443 |
| 26. Errors are made in the transcribed to the electronic /paper order system. | 0,498594 | 0,516766 | 0,567377 | 0,387653 | 0,73325 | 0,347804 |
| 27. Equipment malfunctions or is not set correctly (e.g., IV pump). | 0,315097 | 0,537176 | 0,377078 | 0,475277 | 0,60513 | 0,338944 |
| 28. Nurse is unaware of a known allergy. | 0,173992 | 0,496230 | 0,232072 | 0,462505 | 0,47861 | 0,178250 |
| 29. Patients are off the ward for other care. | 0,175874 | 0,576241 | 0,213130 | 0,537028 | 0,59089 | 0,380262 |
